# Fast-Food Consumption Frequency, Food-Choice Motivations, and Mediterranean Diet Adherence in Young Football Athletes: A Cross-Sectional Study

**DOI:** 10.3390/nu18121850

**Published:** 2026-06-09

**Authors:** Leandro Oliveira, Mariana Salgueiro, Marta Esgalhado

**Affiliations:** 1 CBIOS (Research Center for Biosciences and Health Technologies), ECTS (School of Health Sciences and Technologies), Lusófona University, Campo Grande 376, 1749-024 Lisboa, Portugal; 2 School of Health Sciences and Technologies, Lusófona University, Campo Grande 376, 1749-024 Lisboa, Portugal

**Keywords:** Mediterranean diet, fast food, adolescents, children, athletes, food choice, KIDMED

## Abstract

Background: Dietary behaviours in young athletes are shaped by multiple behavioural, social, and environmental influences, which may contribute to the coexistence of healthy and unhealthy eating patterns. This study aimed to explore factors associated with fast-food consumption frequency and adherence to the Mediterranean diet (MD) among young football athletes. Methods: A cross-sectional study was conducted among 94 male football players aged 10–16 years from a Portuguese football club. Adherence to the MD was assessed using the Mediterranean Diet Quality Index for Children and Adolescents (KIDMED) index. Fast-food consumption patterns and food-choice motivations were evaluated through a structured questionnaire. Anthropometric measurements were obtained using standardised procedures. Correlation, linear regression, and ordinal regression analyses were performed. Results: Most participants demonstrated high adherence to the MD (96.8%), with a median KIDMED score of 12.0 (IQR: 10.0–13.0). Although 88.3% of participants reported consuming fast food, intake frequency was generally low, with 67.0% reporting consumption never or only once per month. Higher fast-food consumption frequency was associated with lower fruit and vegetable intake and higher consumption of sweets and pastries. No significant associations were observed between fast-food consumption frequency and overall MD adherence, Body Mass Index z-score, or fat mass percentage. Higher health-related motivation scores were associated with lower odds of more frequent fast-food consumption (OR = 0.248; *p* = 0.021), whereas greater decision autonomy regarding restaurant choice was associated with higher consumption frequency (OR = 4.036; *p* = 0.010). Conclusions: Young football athletes showed high adherence to the Mediterranean diet despite the presence of fast-food consumption, suggesting that healthy and unhealthy dietary behaviours may coexist within the same population. Behavioural factors, particularly health motivations and food-choice autonomy, appear to influence fast-food consumption independently of overall diet quality and anthropometric status.

## 1. Introduction

The global increase in the consumption of ultra-processed foods, particularly fast food, represents a major and escalating challenge for public health and dietary sustainability. These foods are typically energy-dense, nutritionally imbalanced, and designed for convenience, contributing to the progressive displacement of traditional dietary patterns [1,2]. This dietary transition has been particularly pronounced among younger populations, driven by widespread availability, affordability, and aggressive marketing strategies [3,4]. At a global level, suboptimal dietary patterns are now recognised as one of the leading risk factors for mortality and disease burden, highlighting the urgent need to address unhealthy eating behaviours [5].

Fast-food consumption has been consistently associated with increased risk of overweight, obesity, and cardiometabolic disorders [1,4]. Importantly, experimental evidence supports a causal relationship, demonstrating that diets rich in ultra-processed foods lead to increased energy intake and weight gain under controlled conditions [6]. These effects are particularly concerning in younger populations, as dietary habits established during childhood and adolescence tend to persist into adulthood, reinforcing long-term health risks [7]. Moreover, food choice behaviours in youth are influenced by a complex interaction of individual, social, and environmental factors, including taste preferences, convenience, economic constraints, and social context [8,9].

Adolescence is a critical developmental stage characterised by increasing autonomy in food-related decisions, heightened susceptibility to peer influence, and reduced parental control [10]. In this context, recent research continues to emphasise the predominance of hedonic and convenience-driven factors in shaping dietary behaviours, while health-related motivations tend to play a secondary role [8,11]. Despite the extensive literature on general adolescent populations, evidence focusing specifically on physically active or athletic groups remains limited [12].

In contrast, adherence to the Mediterranean diet has been widely recognised as a cornerstone of healthy and sustainable eating [13,14]. This dietary pattern, characterised by high consumption of plant-based foods, olive oil as the main fat source, and moderate intake of fish and dairy products, has been consistently associated with reduced risk of chronic diseases and improved overall health [15]. High-quality evidence from large-scale trials further supports its protective effects, particularly in cardiovascular disease prevention [16]. More recently, emerging research suggests that adherence to the Mediterranean diet may also support athletic performance, recovery, and body composition, although findings remain heterogeneous [17,18].

Among children and adolescents, higher adherence to the Mediterranean diet has been associated with improved nutritional status, healthier lifestyle behaviours, and better academic performance, although inconsistencies persist across studies due to methodological variability [14,19,20]. In athletic populations, however, evidence remains limited and fragmented, with most studies reporting moderate adherence levels and highlighting the need for further research in this area [21].

Young athletes represent a particularly relevant yet understudied group. Adequate nutrition is essential not only for growth and development but also for optimising performance, recovery, and injury prevention [22]. Nevertheless, recent evidence indicates that many young athletes fail to meet dietary recommendations and may exhibit suboptimal eating behaviours, including insufficient adherence to healthy dietary patterns [23,24]. Nutritional knowledge has been proposed as a relevant factor associated with dietary behaviour in this population, showing a positive association with adherence to the Mediterranean diet [24,25]. At the same time, adolescent athletes face increased nutritional demands due to the combined requirements of growth and high levels of physical activity, which may amplify the consequences of inadequate dietary habits [26,27].

Despite these considerations, the relationship between fast-food consumption and overall dietary quality in young athletes remains poorly understood [28]. Emerging evidence suggests that dietary behaviours may coexist in complex and sometimes contradictory patterns, where healthy and unhealthy practices are simultaneously present, and individuals may simultaneously adhere to healthy dietary patterns while engaging in less healthy practices [29,30]. Furthermore, the factors associated with fast-food consumption in this population, particularly behavioural and contextual factors such as autonomy, social environment, and health motivations, have not been sufficiently explored [9,31]. This gap is particularly relevant given that effective dietary interventions in youth require a comprehensive understanding of both individual and environmental determinants [31].

It is also important to consider that dietary behaviours and Mediterranean diet adherence may vary substantially according to sociocultural and geographic context [9,11,13]. In Mediterranean countries such as Portugal, traditional dietary practices, family eating habits, and food availability may influence eating behaviours differently from non-Mediterranean settings [11,15]. Consequently, findings from international studies may not be directly transferable to Portuguese youth athletic populations, highlighting the importance of context-specific research in this area [18,21].

Addressing these gaps is essential for the development of effective, targeted interventions aimed at improving dietary behaviours in young athletes. Therefore, the aim of this study was to explore factors associated with fast-food consumption frequency and Mediterranean diet adherence among young football athletes.

## 2. Materials and Methods

### 2.1. Study Design and Participants

This observational cross-sectional study aimed to assess adherence to the Mediterranean diet and to explore factors associated with fast-food consumption among young football athletes. The study was reported in accordance with the Strengthening the Reporting of Observational Studies in Epidemiology (STROBE) guidelines, as recommended by the EQUATOR Network, to ensure transparent and comprehensive reporting. A completed STROBE checklist is provided as Supplementary Material (Table S1).

A total of 104 eligible male athletes aged 10 to 16 years, enrolled in youth training and competition teams at Sport União Sintrense (Portugal), were invited to participate. Of these, 94 provided complete data and were included in the final analysis. Inclusion criteria comprised: (i) age between 10 and 16 years; (ii) active registration in the club’s training or competition teams; (iii) residence in Portugal; and (iv) provision of written informed consent by a parent or legal guardian. Participants with incomplete questionnaires or missing key variables were excluded from the analysis.

All athletes were regularly engaged in organised football training and competitive practice; however, none were professional athletes. Therefore, the sample should be interpreted as representative of a specific youth football context rather than of all young athletes.

### 2.2. Data Collection

Data collection was conducted between April and July 2025, during the competitive sports season. Prior to the study, authorization was obtained from the club’s board, and written informed consent was collected from all parents or legal guardians. The study protocol was approved by the Ethics Committee of Universidade Lusófona (reference no. P07-25, 29 April 2025) and conducted in accordance with the Declaration of Helsinki [32]. Participation was voluntary, and all data were collected anonymously and treated confidentially, being used exclusively for research purposes. Each participant was assigned a unique identification code to ensure anonymity.

Participants completed the questionnaire in person, during scheduled anthropometric assessment sessions before training, under the supervision of a researcher to ensure proper completion. The questionnaire completion time ranged between 10 and 15 min.

### 2.3. Instruments and Measures

#### 2.3.1. Dietary Assessment and Questionnaires

A structured questionnaire was used to collect data on sociodemographic characteristics, dietary habits, adherence to the Mediterranean diet, and fast-food consumption patterns, including perceived satisfaction. Adherence to the Mediterranean diet was assessed using the KIDMED index, a validated instrument for children and adolescents. The questionnaire consists of 16 dichotomous (yes/no) items evaluating dietary behaviours aligned with the Mediterranean dietary pattern [33]. Positive responses reflecting healthy dietary habits were scored +1, while responses indicating less healthy behaviours were scored −1, resulting in a total score ranging from −4 to 12. Adherence was categorised as: High adherence (≥8 points); Moderate adherence (4–7 points); Low adherence (≤3 points) [33]. Fast-food consumption was assessed through a structured self-reported questionnaire focused on consumption frequency and contextual behavioural characteristics. Consumption frequency was evaluated using ordinal categories (“never”, “once a month”, “2–3 times per month”, and “1–2 times per week”), reflecting habitual frequency of consumption rather than quantitative intake or dietary records over a defined period. Although fast-food consumption frequency was measured using ordinal categories, the variable was treated as approximately continuous in exploratory regression analyses due to the ordered structure of the categories and the limited number of response levels. This analytical approach was considered exploratory and interpreted cautiously. The questionnaire items were developed based on previous studies assessing fast-food consumption behaviours and food-choice motivations among adolescents and young populations, including Portuguese populations [8].

In addition to consumption frequency, participants were asked about contextual characteristics related to fast-food consumption, including companionship, restaurant choice, and satisfaction with meals consumed at fast-food restaurants. Determinants associated with fast-food consumption were also evaluated using a set of predefined factors, including toy/promotion, price, taste, health concerns, lack of alternatives, quick service, and perceived nutritional value. Each determinant was rated using a five-point Likert scale ranging from “not at all” to “extremely”, reflecting the perceived importance of each factor in influencing food choices.

These items were subsequently grouped into conceptual domains representing sensory motivations (e.g., taste, toy/promotion), convenience motivations (e.g., price, quick service, lack of alternatives), and health-related motivations (e.g., health concerns, nutritional value). Composite scores were calculated for each domain and used to identify factors associated with fast-food consumption.

#### 2.3.2. Anthropometric Assessment

Anthropometric measurements were performed by the same trained researcher prior to training sessions, under standardised conditions (barefoot and wearing light clothing). Body weight was measured using a TANITA BC-601^®^ (Tanita Corporation, Tokyo, Japan) scale, and height was measured using a Seca 217^®^ stadiometer (SECA GmbH & Co. KG, Hamburg, Germany). Three measurements were taken and averaged to ensure accuracy, following national guidelines [34] and international standardised procedures [35]. Skinfold thickness was measured at the tricipital and subscapular sites, with two measurements recorded for each, as recommended for paediatric populations. Body mass index (BMI) was calculated as weight (kg)/height (m^2^) and interpreted according to World Health Organization growth reference standards using age- and sex-specific z-scores [36].

### 2.4. Statistical Analysis

Statistical analyses were performed using Jamovi, version 2.4.14 (The jamovi Project, Sydney, Australia). Continuous variables were assessed for normality using the Shapiro–Wilk test and are presented as mean and standard deviation (SD) when normally distributed, or as median and interquartile range (IQR) when non-normally distributed. Categorical variables are presented as absolute and relative frequencies (*n*, %).

Comparisons between age groups (childhood vs. adolescence) were performed using the independent samples *t*-test for normally distributed variables and the Mann–Whitney U test for non-normally distributed variables. Associations between categorical variables were assessed using the Chi-square test. When expected cell counts were below 5, including situations with sparse tables, Fisher’s exact test was applied instead. Differences according to fast-food consumption frequency were analysed using one-way ANOVA or the Kruskal–Wallis test depending on data distribution.

Associations between continuous or ordinal variables were evaluated using Spearman’s correlation coefficients.

Determinants of fast-food consumption were grouped into three domains: sensory motivations (taste, toy), convenience motivations (price, quick service, lack of alternatives), and health motivations (health concerns, nutritional value). Each item was originally measured on a five-point Likert scale (“not at all”, “a little”, “moderately”, “a lot”, “extremely”) and converted into continuous scores (0, 0.25, 0.5, 0.75, 1). Scores within each domain were summed to generate composite variables representing each motivational domain.

To examine factors associated with fast-food consumption frequency, an ordinal logistic regression model was performed including health motivations, age, and the variable indicating who usually chooses the restaurant as independent variables. Results are presented as odds ratios (OR) with 95% confidence intervals (95% CI).

Additionally, a linear regression analysis was conducted to explore potential factors associated with the KIDMED score, including fast-food consumption frequency, age, and BMI z-score as independent variables. Regression coefficients (B) and 95% CI were reported. Statistical significance was set at *p* ≤ 0.05.

## 3. Results

A total of 94 youth football players aged 10–16 years participated in the study, including 54 children and 40 adolescents (Table 1). Significant differences between age groups were observed for age and educational level (both *p* < 0.001). Most participants resided in Sintra, and no significant differences were observed between groups regarding residence or playing position.

Anthropometric assessment showed that children presented slightly higher BMI z-scores and a higher prevalence of overweight/obesity compared with adolescents. No significant differences were observed between age groups for skinfold sum or fat mass percentage.

The detailed outcomes of the KIDMED questionnaire stratified by age group are presented in the Supplementary Material (Table S2). Overall, participants demonstrated high adherence to the Mediterranean diet, with no significant differences in total KIDMED scores between children and adolescents. Significant age-related differences were observed for fruit and vegetable consumption, which were generally higher among children, whereas adolescents more frequently reported consuming pastries or baked goods at breakfast. No significant differences were observed for the remaining dietary behaviours.

Fast-food consumption was generally infrequent, with most participants reporting consumption once per month or less, and no significant differences were observed between age groups (Table 2). Among those who consumed fast food, meals were predominantly consumed with family members. However, adolescents more frequently reported consuming fast food with friends and independently choosing the restaurant compared with children (both *p* < 0.001). Most participants also reported being satisfied or very satisfied with meals consumed at fast-food restaurants.

The factors associated with participants’ food choices when consuming fast-food meals are presented in the Supplementary Material (Table S3). Taste and health concerns emerged as the most influential determinants of food choice. Adolescents attributed greater importance to taste-, convenience-, and price-related factors compared with children. Significant differences according to fast-food consumption frequency were observed only for health concerns and quick service.

Participants’ anthropometric characteristics and adherence to the Mediterranean diet were generally similar across fast food consumption frequency groups (Table 3). No significant differences were observed for age, body composition indicators, or KIDMED scores. A significant difference was identified only for BMI z-score, with participants consuming fast-food 2–3 times per month presenting higher values compared with the remaining groups.

Correlation analyses were performed to explore relationships between age, diet quality, fast-food consumption frequency, and body composition (Table 4). A significant negative association between age and BMI z-score (r = −0.270, *p* = 0.008) indicated that older participants tended to have slightly lower BMI z-scores. Fat mass was strongly positively correlated with BMI z-score (r = 0.512, *p* < 0.001), reflecting the close relationship between overall adiposity and BMI in this sample. No other correlations reached statistical significance.

Correlations between individual KIDMED questionnaire items, determinants of fast-food consumption, and fast-food consumption frequency are presented in the Supplementary Material (Table S4). Higher fast-food consumption frequency was associated with lower fruit and vegetable intake and higher consumption of sweets, pastries, and dairy products at breakfast. No significant correlations were identified between fast-food consumption frequency and the grouped motivational domains.

Ordinal regression analysis identified health-related motivations and restaurant-choice autonomy as significant factors associated with fast-food consumption frequency (Table 5). Higher health-related motivation scores were associated with lower odds of more frequent fast-food consumption, whereas participants who independently chose the restaurant were more likely to report higher consumption frequency. No significant associations were observed for sensory motivations, convenience-related motivations, age, or BMI z-score.

Exploratory linear regression analysis showed no significant associations between KIDMED score and fast-food consumption frequency, age, or BMI z-score, with all confidence intervals crossing zero (all *p* > 0.05).

## 4. Discussion

The present study explored Mediterranean diet adherence, fast-food consumption frequency, and the behavioural factors associated with fast-food intake among young football athletes. Overall, participants demonstrated high adherence to the Mediterranean diet, with almost all athletes classified as having high KIDMED scores. At the same time, fast-food consumption remained present in most participants, although generally at low frequencies, with the majority reporting consumption once per month. These findings suggest that healthy and unhealthy dietary behaviours may coexist within the same dietary pattern, reinforcing the complexity of eating behaviours during childhood and adolescence.

Adherence to the Mediterranean diet observed in this study was notably higher than what is commonly reported in youth athletic populations, where moderate adherence tends to predominate. Although the Mediterranean diet is widely recognised for its benefits in health and sports performance, including improved recovery, reduced inflammation, and enhanced physical capacity, evidence suggests that adherence remains inconsistent across youth athletic populations [18,37,38]. Furthermore, recent evidence suggests that the current body of literature on Mediterranean diet adherence in athletes is still limited and methodologically heterogeneous, which may partly explain the variability observed across studies [18].

Importantly, our findings support the notion that participation in organised sport alone does not guarantee healthier dietary behaviours. Despite the high adherence to the Mediterranean diet observed in this sample, certain suboptimal patterns persisted, including a relatively high consumption of sweets and pastries and lower intake of a second daily portion of fruit. Despite the increased nutritional demands associated with growth, development, and training, the present findings, together with the previous literature, suggest that some young athletic populations may demonstrate suboptimal dietary habits, including the consumption of energy-dense, nutrient-poor foods [23,26]. This pattern may reflect the complex interplay between physiological needs and behavioural drivers, where convenience, palatability, and environmental exposure often outweigh nutritional considerations [39].

The very high proportion of participants classified as having high adherence to the Mediterranean diet should be interpreted with caution. Although this result may reflect the cultural proximity of the sample to the Mediterranean dietary pattern and possible family-based dietary practices, it may also indicate an overestimation of diet quality. The KIDMED index is a brief self-reported screening tool and, similarly to other dietary self-report instruments, may be influenced by recall bias and social desirability bias, particularly in supervised administration contexts [40]. This issue is particularly relevant because high KIDMED adherence coexisted with reported fast-food consumption, pastries at breakfast, and frequent intake of sweets among part of the sample. Previous studies have also suggested that healthy and unhealthy dietary behaviours may coexist within the same individuals, particularly during adolescence [9,30]. Therefore, the findings should not be interpreted as evidence of uniformly optimal dietary behaviour, but rather as an indication of the complexity and multidimensional nature of eating behaviours in young populations. The findings should also be interpreted within the specific sociocultural and sporting context of Portuguese youth football, where family eating practices, Mediterranean food traditions, and local food environments may influence dietary behaviours differently from other athletic populations.

The coexistence of Mediterranean diet adherence and fast-food consumption identified in this study aligns with emerging perspectives that dietary behaviours should not be interpreted as mutually exclusive [30].

Recent evidence in youth sport populations further supports the complexity of lifestyle and dietary behaviours among young athletes [21,23,24]. Although physically active individuals are often expected to present healthier eating habits, participation in organised sport alone does not necessarily protect against unhealthy food choices or other suboptimal lifestyle behaviours [23,24]. In this context, recent studies have highlighted that Mediterranean diet adherence may coexist with less healthy eating patterns and lifestyle characteristics, reinforcing the importance of interpreting diet quality within a broader behavioural framework [30,41]. Similarly, evidence from collegiate athletes suggests that lifestyle-related factors such as sleep quality, recovery habits, and behavioural routines may vary substantially even among physically active populations, highlighting the multidimensional nature of health-related behaviours in athletes [42]. These findings support the view that adherence to the Mediterranean diet should not be interpreted as evidence of uniformly healthy behaviours, particularly during adolescence, when food choices are strongly influenced by autonomy, peer interactions, convenience, and environmental exposure [9,10,31].

The absence of a clear association between KIDMED scores and fast-food frequency suggests that overall diet quality and specific eating behaviours may function as partly independent dimensions. In this context, adolescents may simultaneously adhere to certain healthy dietary components while engaging in unhealthy eating practices, reflecting a dynamic and context-dependent decision-making process [10,43]. This duality highlights the limitations of dichotomous interpretations of dietary quality and underscores the importance of considering overall dietary patterns [9].

Behavioural and contextual factors appear to be associated with fast-food consumption patterns in this sample. Health-related motivations and decision autonomy emerged as significant factors, suggesting that behavioural and contextual elements may be more relevant than sensory or convenience-related drivers in this population. This finding aligns with previous research indicating that food choices in adolescents are influenced by complex decision-making processes that extend beyond individual preferences [8,44]. Furthermore, previous evidence suggests that nutritional knowledge may influence dietary behaviours; however, its effect appears to be modest and insufficient to ensure adherence to healthy dietary patterns [24]. These findings may suggest that nutritional knowledge alone is not necessarily associated with healthier dietary behaviours [45].

From a theoretical perspective, these findings may be interpreted within a broader socioecological framework of eating behaviour, in which food choices emerge from the interaction between individual motivations, social influences, environmental accessibility, and behavioural autonomy [46]. In adolescence, increasing decision-making autonomy may simultaneously favour independent food choices and greater exposure to convenience-oriented eating environments, particularly in social settings involving peers [10,31]. Conversely, stronger health-related motivations may reflect greater internalisation of health values, family influence, or nutritional awareness, which could partially explain their association with lower fast-food consumption frequency in this sample. These observations reinforce the multidimensional nature of dietary behaviours and highlight the limitations of approaches focused exclusively on nutritional knowledge transmission [9,11].

Anthropometric indicators, including BMI and skinfold thickness, were explored as potential correlates of dietary behaviour. Although clear associations may not have emerged, this is consistent with the recent literature indicating that the relationship between body composition and dietary patterns in adolescents is complex and often inconclusive, particularly in cross-sectional studies [47]. Additionally, developmental factors such as age and sex may influence dietary behaviours, reflecting differences in autonomy, social influences, and health awareness during adolescence [10,47]. From a broader perspective, the findings suggest that nutritional interventions in youth sport settings may benefit from a more comprehensive behavioural and environmental approach. While the Mediterranean diet has demonstrated potential benefits for both health and performance, including improvements in aerobic capacity and strength outcomes [17,19], its adoption in young populations remains limited. This suggests that traditional education-based approaches may be insufficient, and that more comprehensive strategies addressing environmental, social, and behavioural determinants are required [46].

The practical implications of this study are particularly relevant for coaches, health professionals, and policymakers. Given that adolescence represents a critical period for the establishment of long-term dietary habits, targeted interventions in sports contexts may provide a valuable opportunity to promote healthier eating behaviours. However, such interventions should be multidimensional, integrating nutrition education with environmental modifications and behavioural support, in line with contemporary models of dietary behaviour change.

### Limitations and Future Research

This study has several limitations that should be acknowledged. First, the cross-sectional design precludes causal inference, and the observed associations should therefore be interpreted cautiously. Second, the sample was recruited from a single football club in Portugal, which limits the external validity and generalisability of the findings to broader populations of young athletes with different cultural, socioeconomic, and sporting backgrounds.

Third, dietary behaviours and fast-food consumption frequency were assessed using self-reported measures, potentially introducing recall bias and social desirability bias. This issue may have been further amplified by the supervised administration context, which could have contributed to the reporting of healthier dietary behaviours and to the exceptionally high KIDMED adherence observed in the sample. In addition, although the KIDMED index is a widely used screening instrument in paediatric populations, it provides a relatively simplified assessment of diet quality and may not fully capture the complexity of eating behaviours.

Another limitation relates to the assessment of fast-food consumption. The questionnaire focused primarily on consumption frequency and food-choice motivations, without collecting detailed information regarding portion size, specific food items consumed, nutritional composition, or overall dietary intake. Furthermore, the questionnaire used to assess fast-food-related behaviours was not formally validated, and fast-food consumption was assessed through broad ordinal frequency categories rather than quantitative dietary assessment methods. These categories may not fully reflect meaningful variability in consumption patterns. Additionally, although ordinal frequency categories were treated as approximately continuous in exploratory regression analyses, this approach may not fully capture the non-linear nature of consumption behaviours and should therefore be interpreted cautiously.

Moreover, although several associations reached statistical significance, most effect sizes were modest, indicating limited practical impact. In addition, given the sample size, the regression results should be considered exploratory and interpreted as hypothesis-generating rather than confirmatory.

Future studies should adopt longitudinal and multicentre designs, include larger and more diverse athletic populations, and incorporate more comprehensive and validated dietary assessment methods to better understand the complexity of dietary behaviours in young athletes.

## 5. Conclusions

This study provides preliminary insight into dietary behaviours among young football athletes within a Portuguese football club context, highlighting the coexistence of high Mediterranean diet adherence and the presence of fast-food consumption. The findings suggest that engagement in sport does not necessarily ensure optimal dietary habits, reinforcing the need to address behavioural and environmental determinants of food choice in this population. Fast-food consumption appears to coexist with several healthier dietary behaviours, reflecting the complexity of eating behaviours during adolescence. Given the increased nutritional demands associated with growth and physical activity, promoting healthier dietary behaviours may be particularly relevant in young athletes. Interventions should move beyond knowledge-based approaches and incorporate strategies targeting accessibility, social influences, and behavioural habits. Despite its limitations, this study contributes to the current understanding of dietary patterns in youth sport settings and highlights the need for further research using larger and more diverse samples, as well as longitudinal designs, to better understand the determinants of dietary behaviours in this population.

## Figures and Tables

**Table 1:** Sociodemographic characteristics of the athletes by age groups.

	Total (*n* = 94)	Childhood (*n* = 54)	Adolescence (*n* = 40)	*p*-Value
Age (years), median (IQR)	12.0 (10.0; 13.8)	10.0 (10.0; 11.0)	14.0 (13.0; 14.3)	<0.001 ^a^
Educational level, *n* (%)				
Primary education (3rd–4th grade)	21 (22.3)	21 (38.9)	0 (0.0)	<0.001 ^b^
Lower secondary education (5th–6th grade)	32 (34.0)	31 (57.4)	1 (2.5)	
Middle secondary education (7th–9th grade)	34 (36.2)	2 (3.7)	32 (80.0)	
Upper secondary education (10th–11th grade)	7 (7.4)	0 (0.0)	7 (17.5)	
Residence, *n* (%)				
Cascais	7 (7.4)	6 (11.1)	1 (2.5)	0.118 ^b^
Mafra	5 (5.3)	3 (5.6)	2 (5.0)	
Oeiras	10 (10.6)	3 (5.6)	7 (17.5)	
Sintra	72 (76.6)	42 (77.8)	30 (75.0)	
Playing position, *n* (%)				
Goalkeeper	13 (13.8)	5 (9.3)	8 (20.0)	0.277 ^c^
Defender	25 (26.6)	17 (31.5)	8 (20.0)	
Midfielder	18 (19.1)	7 (13.0)	11 (27.5)	
Winger	21 (22.3)	13 (24.1)	8 (20.0)	
Forward	17 (18.1)	13 (24.1)	4 (10.0)	
BMI z-score, median (IQR)	0.3 (−0.15; 0.8)	0.41 (−0.1; 1.1)	0.12 (−0.5; 0.7)	0.035 ^a^
Nutritional status, *n* (%)				
Percentile < 5: Underweight	21 (22.3)	2 (3.7)	3 (7.5)	0.015 ^b^
Percentile ≥ 5 e < 85: Normal weight	71 (75.5)	36 (66.7)	35 (87.5)	
Percentile ≥ 85 e < 95: Overweight	14 (14.9)	12 (22.2)	2 (5.0)	
Percentile ≥ 95: Obesity	4 (4.3)	4 (7.4)	0 (0.0)	
Σ Skinfolds(mm), mean (SD)	18.7 (2.4)	18.7 (1.8)	18.6 (3.1)	0.874 ^d^
Fat mass (%), mean (SD)	18.1 (2.2)	18.1 (1.7)	18.0 (2.8)	0.792 ^d^

Data are presented as median (interquartile range, IQR) and mean (standard deviation, SD) for continuous variables, and absolute values (percentage) for categorical variables. Group comparisons were assessed using ^a^ Mann–Whitney U test, ^b^ Fisher’s exact test, ^c^ Chi-square test or ^d^ *T*-test, as appropriate. Abbreviations: BMI, body mass index.

**Table 2:** Patterns of fast-food consumption by age groups.

	Total (*n* = 94)	Childhood (*n* = 54)	Adolescence (*n* = 40)	*p*-Value
How often do you consume fast-food meals?				
Never	11 (11.7)	8 (14.8)	3 (7.5)	0.363 ^a^
Once a month	52 (55.3)	32 (59.3)	20 (50.0)	
2–3 times per month	22 (23.4)	10 (18.5)	12 (30.0)	
1–2 times per week	9 (9.6)	4 (7.4)	5 (12.5)	
	Total (*n* = 83)	Childhood (*n* = 46)	Adolescence (*n* = 37)	*p*-value
Do you usually consume fast-food meals with your family?				
Yes	80 (96.4)	44 (95.7)	36 (97.3)	1.000 ^b^
No	3 (3.6)	2 (4.3)	1 (2.7)	
Do you usually consume fast-food meals with your friends?				
Yes	51 (61.4)	20 (43.5)	31 (83.8)	<0.001 ^a^
No	32 (38.6)	26 (56.5)	6 (16.2)	
Which restaurant do you visit most frequently?				
McDonald’s	57 (68.7)	33 (71.7)	24 (64.9)	0.379 ^a^
Burger King	13 (15.7)	5 (10.9)	8 (21.6)	
Other	13 (15.7)	8 (17.4)	5 (13.5)	
Do you usually choose the children’s menu?				
Yes	9 (10.8)	7 (15.2)	2 (5.4)	0.287 ^b^
No	74 (89.2)	39 (84.8)	35 (94.6)	
Who usually chooses the restaurant?				
Myself	41 (49.4	11 (23.9)	30 (81.1)	<0.001 ^a^
Family	42 (50.6)	35 (76.1)	7 (18.9)	
What is your level of satisfaction with the meal consumed at fast-food restaurants?				
Dissatisfied	1 (1.2)	0 (0.0)	1 (2.7)	0.070 ^b^
Satisfied	57 (68.7)	28 (60.9)	29	
Very satisfied	25 (30.1)	18 (39.1)	7 (18.9)	

Data are presented as absolute values (percentage). Group comparisons were assessed using ^a^ Chi-square test, ^b^ Fisher’s exact test or, as appropriate.

**Table 3:** Sociodemographic, anthropometric, and Mediterranean diet adherence characteristics by fast-food consumption frequency.

	Fast-Food Consumption Frequency				
	Never (*n* = 11)	Once a Month (*n* = 52)	2–3 Times per Month (*n* = 22)	1–2 Times per Week (*n* = 9)	*p* Value
Age, median (IQR)	11.4 (10.0; 12.5)	12.0 (10.0; 14.0)	11.9 (10.0; 13.0)	12.0 (10.0; 14.0)	0.863 ^a^
BMI z-score, mean (SD)	−0.3 (0.8)	0.4 (0.8)	0.5 (0.7)	−0.5 (1.4)	0.034 ^b^
Σ skinfolds, mean (SD)	18.0 (2.7)	18.3 (2.4)	19.6 (2.4)	18.9 (2.1)	0.207 ^b^
Fat mass %, mean (SD)	17.5 (2.5)	17.8 (2.2)	18.9 (2.2)	18.3 (1.9)	0.200 ^b^
KIDMED score, median (IQR)	10.6 (10.0; 12.5)	11.3 (10.0; 13.0)	11.5 (10.0; 13.0)	10.9 (10.0; 12.0)	0.753 ^a^

Data are presented as median (interquartile range, IQR) and mean (standard deviation, SD). Group comparisons were assessed using ^a^ Kruskal–Wallis test or ^b^ ANOVA test, as appropriate. Abbreviations: BMI, body mass index.

**Table 4:** Correlations among age, Mediterranean diet adherence, fast-food consumption frequency, and body composition.

	Age	KIDMED Score	Fast-Food Frequency	BMI z Score	Fat Mass
Age	-				
KIDMED score	−0.019 (0.853)	-			
Fast-food frequency	0.048 (0.645)	0.009 (0.928)	-		
BMI z-score	−0.270 (0.008)	0.047 (0.650)	0.088 (0.400)	-	
Fat mass	−0.099 (0.341)	0.163 (0.116)	0.188 (0.070)	0.512 (<0.001)	-

Values are presented as Spearman correlation coefficients, with corresponding *p*-values in parentheses. Abbreviations: BMI, body mass index.

**Table 5:** Ordinal regression analysis of factors associated with fast-food consumption frequency.

	OR	95% CI	*p*-Value
Health motivations	0.248	0.072–0.795	0.021
Age	0.988	0.747–1.303	0.932
Restaurant choice (myself-family)	4.036	1.423–12.142	0.010

Values are presented as odds ratios (OR) with 95% confidence intervals (CI).

## Data Availability

Data are contained within the article.

## Supplementary Materials

The following supporting information can be downloaded at: https://www.mdpi.com/article/10.3390/nu18121850/s1, Table S1. STROBE Statement—Checklist of items that should be included in reports of cross-sectional studies. Table S2. KIDMED questionnaire outcomes by age groups. Table S3. Factors associated with fast-food consumption by age groups and fast-food consumption frequency. Table S4. Correlations among KIDMED questionnaire outcomes and determinants for fast-food consumption and fast-food consumption frequency.

## Abbreviations

The following abbreviations are used in this manuscript:

BMI	Body Mass Index
CI	Confidence Interval
IQR	Interquartile Range
KIDMED	Mediterranean Diet Quality Index for children and adolescents
MD	Mediterranean Diet
OR	Odds Ratio
SD	Standard Deviation
STROBE	Strengthening the Reporting of Observational Studies in Epidemiology
WHO	World Health Organization
