# Supplementary material for: Fast-Food Consumption Frequency, Food-Choice Motivations, and Mediterranean Diet Adherence in Young Football Athletes: A Cross-Sectional Study
Source: Nutrients. 2026 Jun 9;18(12):1850. doi: 10.3390/nu18121850 (PMC13304575; doi:10.3390/nu18121850)
Supplement: Supplementary file 1 [file nutrients-18-01850-s001.zip › nutrients-4297482-supplementary.pdf]

Table S1. STROBE Statement: Checklist of items that should be included in reports of *cross-sectional studies*

	Item No	Recommendation	Page No
**Title and abstract**	1	(*a*) Indicate the study's design with a commonly used term in the title or the abstract	1
		(*b*) Provide in the abstract an informative and balanced summary of what was done and what was found	1
**Introduction**			
Background/rationale	2	Explain the scientific background and rationale for the investigation being reported	2
Objectives	3	State specific objectives, including any prespecified hypotheses	3
**Methods**			
Study design	4	Present key elements of study design early in the paper	4
Setting	5	Describe the setting, locations, and relevant dates, including periods of recruitment, exposure, follow-up, and data collection	4-5
Participants	6	(*a*) Give the eligibility criteria, and the sources and methods of selection of participants	5-6
Variables	7	Clearly define all outcomes, exposures, predictors, potential confounders, and effect modifiers. Give diagnostic criteria, if applicable	5-7
Data sources/ measurement	8*	For each variable of interest, give sources of data and details of methods of assessment (measurement). Describe comparability of assessment methods if there is more than one group	*8*
Bias	9	Describe any efforts to address potential sources of bias	14-15
Study size	10	Explain how the study size was arrived at	4
Quantitative variables	11	Explain how quantitative variables were handled in the analyses. If applicable, describe which groupings were chosen and why	7
Statistical methods	12	(*a*) Describe all statistical methods, including those used to control for confounding	7
		(*b*) Describe any methods used to examine subgroups and interactions	7
		(*c*) Explain how missing data were addressed	4,7
		(*d*) If applicable, describe analytical methods taking account of sampling strategy	7
		(*e*) Describe any sensitivity analyses	N/A
**Results**			
Participants	13*	(a) Report numbers of individuals at each stage of study—eg numbers potentially eligible, examined for eligibility, confirmed eligible, included in the study, completing follow-up, and analysed	4, 8-9
		(b) Give reasons for non-participation at each stage	4
		(c) Consider use of a flow diagram	N/A
Descriptive data	14*	(a) Give characteristics of study participants (eg demographic, clinical, social) and information on exposures and potential confounders	8-12
		(b) Indicate number of participants with missing data for each variable of interest	N/A
Outcome data	15*	Report numbers of outcome events or summary measures	8-13

Main results	16	(*a*) Give unadjusted estimates and, if applicable, confounder-adjusted estimates and their precision (eg, 95% confidence interval). Make clear which confounders were adjusted for and why they were included	13
		(*b*) Report category boundaries when continuous variables were categorized	5,7,13
		(*c*) If relevant, consider translating estimates of relative risk into absolute risk for a meaningful time period	N/A
Other analyses	17	Report other analyses done—eg analyses of subgroups and interactions, and sensitivity analyses	8-13
**Discussion**			
Key results	18	Summarise key results with reference to study objectives	14-15
Limitations	19	Discuss limitations of the study, taking into account sources of potential bias or imprecision. Discuss both direction and magnitude of any potential bias	15
Interpretation	20	Give a cautious overall interpretation of results considering objectives, limitations, multiplicity of analyses, results from similar studies, and other relevant evidence	14-16
Generalisability	21	Discuss the generalisability (external validity) of the study results	15
**Other information**			
Funding	22	Give the source of funding and the role of the funders for the present study and, if applicable, for the original study on which the present article is based	16

*Give information separately for exposed and unexposed groups.

Note: An Explanation and Elaboration article discusses each checklist item and gives methodological background and published examples of transparent reporting. The STROBE checklist is best used in conjunction with this article (freely available on the Web sites of PLoS Medicine at http://www.plosmedicine.org/, Annals of Internal Medicine at http://www.annals.org/, and Epidemiology at http://www.epidem.com/). Information on the STROBE Initiative is available at www.strobe-statement.org.

Table S2. KIDMED questionnaire outcomes by age groups.

Variable, n (%)	Total (n=94)	Childhood (n=54)	Adolescence (n=40)	P-value
Q1- Do you eat a piece of fruit or drink a natural fruit juice every day?				
Yes	91 (96.8)	52 (96.3)	39 (97.5)	
No	3 (3.2)	2 (3.7)	1 (2.5)	1.000 a
Q2- Do you eat a second piece of fruit every day?				
Yes	76 (80.9)	50 (92.6)	26 (65.0)	**0.001** a
No	18 (19.1)	4 (7.4)	14 (35.0)	
Q3- Do you regularly eat fresh or cooked vegetables at least once a day?				
Yes	87 (92.6)	49 (90.7)	38 (95.0)	0.695 a
No	7 (7.4)	5 (9.3)	2 (5.0)	
Q4- Do you eat fresh or cooked vegetables more than once a day?				
Yes	51 (54.3)	40 (74.1)	11 (27.5)	
No	43 (45.7)	14 (25.9)	29 (72.5)	< **0.001** a
Q5- Do you eat fish (e.g., hake, sardines, octopus, shrimp) 2–3 times per week?				
Yes	71 (75.5)	41 (75.9)	30 (75.0)	0.918 a
No	23 (24.5)	13 (24.1)	10 (25.0)	
Q6- Do you eat at "fast-food" restaurants once a week or more?				
Yes	13 (13.8)	6 (11.1)	7 (17.5)	
No	81 (86.2)	48 (88.9)	33 (82.5)	0.375 a
Q7- Do you like and eat legumes (beans, peas, chickpeas, broad beans, lentils) more than once a week?				
Yes	75 (79.8)	46 (85.2)	29 (72.5)	
No	19 (20.2)	8 (14.8)	11 (27.5)	0.130 a
Q8- Do you eat pasta or rice almost every day (≥5 times/week)?				
Yes	85 (90.4)	47 (87.0)	38 (95.0)	
No	9 (9.6)	7 (13.0)	2 (5.0)	0.293 a
Q9- Do you eat cereals or cereal products (e.g., oats, bread) for breakfast?				
Yes	88 (93.6)	50 (92.6)	38 (95.0)	
No	6 (6.4)	4 (7.4)	2 (5.0)	1.000 a
Q10- Do you eat nuts (e.g., walnuts, almonds, hazelnuts) 2–3 times per week				
Yes	55 (58.5)	28 (51.9)	27 (67.5)	
No	39 (41.5)	26 (48.1)	13 (32.5)	0.128 a
Q11- Do you use olive oil at home?				
Yes	86 (91.5)	48 (88.9)	38 (95.0)	

No	8 (8.5)	6 (11.1)	2 (5.0)	0.460 a
Q12- Do you have breakfast every day?				
Yes	86 (91.5)	50 (92.6)	36 (90.0)	
No	8 (8.5)	4 (7.4)	4 (10.0)	0.719 a
Q13- Do you eat dairy products (yogurt, milk, etc.) at breakfast?				
Yes	80 (85.1)	48 (88.9)	32 (80.0)	
No	14 (14.9)	6 (11.1)	8 (20.0)	0.231 a
Q14- Do you eat pastries or baked goods at breakfast?				
Yes	33 (35.1)	14 (25.9)	19 (47.5)	
No	61 (64.9)	40 (74.1)	21 (52.5)	**0.030** a
Q15- Do you eat 2 yogurts and/or 40g of cheese per day?				
Yes	58 (61.7)	36 (66.7)	22 (55.0)	
No	36 (38.3)	18 (33.3)	18 (45.0)	0.250 a
Q16- Do you eat sweets or treats several times a day?				
Yes	19 (20.2)	11 (20.4)	8 (20.0)	
No	75 (79.8)	43 (79.6)	32 (80.0)	0.965 a
Total KIDMED score, median (IQR)	12.0 (10.0;13.0)	12.0 (10.0;13.0)	11.5 (10.0;13.0)	0.648 c
Adherence to Mediterranean diet, n (%)				
Low/Moderate	3 (3.2)	1 (1.9)	2 (5.0)	0.178 b
High	91 (96.8)	53 (98.1)	38 (95.0)	

Data are presented as absolute values (percentage) for categorical variables and median (interquartile range, IQR) for continuous variables. Group comparisons were assessed using, a Chi-square test, b Fisher's exact test, or c Mann–Whitney U test, as appropriate.

Table S3. Factors associated with fast-food consumption by age groups and fast-food consumption frequency.

	Total (n=83)	Childhood (n=46)	Adolescence (n=37)	P-value	Fast-food consumption frequency			
					Once a month (n=52)	2–3 times per month (n=22)	1–2 times per week (n=9)	P-value
Toy								
Not at all	78 (94.0)	41 (89.1)	37 (100.0)	0.333 a	50 (96.2)	20 (90.9)	8 (88.9)	0.069 a
A little	2 (2.4)	2 (4.3)	0 (0.0)		2 (3.8)	0 (0.0)	0 (0.0)	
Moderately	2 (2.4)	2 (4.3)	0 (0.0)		0 (0.0)	2 (9.1)	0 (0.0)	
A lot	1 (1.2)	1 (2.2)	0 (0.0)		0 (0.0)	0 (0.0)	1 (11.1)	
Price								
Not at all	29 (34.9)	20 (43.5)	9 (24.3)	**<0.001** b	20 (38.5)	5 (22.7)	4 (44.4)	0.375 a
A little	6 (7.2)	5 (10.9)	1 (2.7)		3 (5.8)	2 (9.1)	1 (11.1)	
Moderately	14 (16.9)	11 (23.9)	3 (8.1)		6 (11.5)	6 (27.3)	2 (22.2)	
A lot	16 (19.3)	7 (15.2)	9 (24.3)		9 (17.3)	5 (22.7)	2 (22.2)	
Extremely	18 (21.7)	3 (6.5)	15 (40.5)		14 (26.9)	4 (18.2)	0 (0.0)	
Taste								
Not at all	1 (1.2)	1 (2.2)	0 (0.0)	**0.005** a	0 (0.0)	0 (0.0)	1 (11.1)	0.315 a
A little	1 (1.2)	1 (2.2)	0 (0.0)		1 (1.9)	0 (0.0)	0 (0.0)	
Moderately	5 (6.0)	5 (10.9)	0 (0.0)		4 (7.7)	1 (4.5)	0 (0.0)	
A lot	29 (34.9)	20 (43.5)	9 (24.3)		16 (30.8)	11 (50.0)	2 (22.2)	
Extremely	47 (56.6)	19 (41.3)	28 (75.7)		3 (5.8)	10 (45.5)	6 (66.7)	
Health Concerns								
Not at all	2 (2.4)	1 (2.2)	1 (2.7)	0.263 a	0 (0.0)	0 (0.0)	2 (22.2)	**0.008** a
A little	3 (3.6)	2 (4.3)	1 (2.7)		1 (1.9)	1 (4.5)	1 (11.1)	
Moderately	7 (8.4)	6 (13.0)	1 (2.7)		5 (9.6)	1 (4.5)	1 (11.1)	
A lot	35 (42.2)	21 (45.7)	14 (37.8)		20 (38.5)	14 (63.6)	1 (11.1)	
Extremely	36 (43.4)	16 (34.8)	20 (54.1)		26 (50.0)	6 (27.3)	4 (44.4)	

Lack of Alternatives								
Not at all	18 (21.7)	15 (32.6)	3 (8.1)		10 (19.2)	4 (18.2)	4 (44.4)	
A little	7 (8.4)	5 (10.9)	2 (5.4)		5 (9.6)	2 (9.1)	0 (0.0)	
Moderately	15 (18.1)	11 (23.9)	4 (10.8)	**0.002** b	9 (17.3)	5 (22.7)	1 (11.1)	0.378 a
A lot	15 (18.1)	7 (15.2)	8 (21.6)		7 (13.5)	7 (31.8)	1 (11.1)	
Extremely	28 (33.7)	8 (17.4)	20 (54.1)		21 (40.4)	4 (18.2)	3 (33.3)	
Quick Service								
Not at all	4 (4.8)	2 (4.3)	2 (5.4)		0 (0.0)	1 (4.5)	3 (33.3)	
A little	6 (7.2)	5 (10.9)	1 (2.7)		3 (5.8)	2 (9.1)	1 (11.1)	**0.050** a
Moderately	16 (19.3)	13 (28.3)	3 (8.1)	**0.027** a	12 (23.1)	4 (18.2)	0 (0.0)	
A lot	25 (30.1)	14 (30.4)	11 (29.7)		16 (30.8)	7 (31.8)	2 (22.2)	
Extremely	32 (38.6)	12 (26.1)	20 (54.1)		21 (40.4)	8 (36.4)	3 (33.3)	
Nutritional Value								
Not at all	3 (3.6)	2 (4.3)	1 (2.7)		1 (1.9)	1 (4.5)	1 (11.1)	
A little	10 (12.0)	9 (19.6)	1 (2.7)		7 (13.5)	2 (9.1)	1 (11.1)	
Moderately	15 (18.1)	14 (30.4)	1 (2.7)	**<0.001**a	7 (13.5)	5 (22.7)	3 (33.3)	0.577 a
A lot	30 (36.1)	14 (30.4)	16 (43.2)		19 (36.5)	9 (40.9)	2 (22.2)	
Extremely	25 (30.1)	7 (15.2)	18 (48.6)		18 (34.6)	5 (22.7)	2 (22.2)	

Data are presented as absolute values (percentage). Group comparisons were assessed using, a Fisher’s exact test or b Chi-square test, as appropriate.

Table S4. Correlations among KIDMED questionnaire outcomes and determinants for fast-food consumption and fast-food consumption frequency.

**KIDMED questionaire**	Fast-food frequency
Q1- Do you eat a piece of fruit or drink a natural fruit juice every day?	-0.031 (0.768)
Q2- Do you eat a second piece of fruit every day?	-0.202 (**0.050**)
Q3- Do you regularly eat fresh or cooked vegetables at least once a day?	-0.180 (0.082)
Q4- Do you eat fresh or cooked vegetables more than once a day?	-0.245 (**0.017**)
Q5- Do you eat fish (e.g., hake, sardines, octopus, shrimp) 2–3 times per week?	-0.124 (0.233)
Q6- Do you visit fast-food restaurants once or more per week?%, (n)	0.558 (**<0.001**)
Q7- Do you like and eat legumes (beans, peas, chickpeas, broad beans, lentils) more than once a week?	-0.051 (0.627)
Q8- Do you eat pasta or rice almost every day (≥5 times/week)?	-0.162 (0.118)
Q9- Do you eat cereals or cereal products (e.g., oats, bread) for breakfast?	-0.062 (0.552)
Q10- Do you eat nuts (e.g., walnuts, almonds, hazelnuts) 2–3 times per week	-0.061 (0.560)
Q11- Do you use olive oil at home?	0.001 (0.994)
Q12- Do you have breakfast every day?	-0.081 (0.438)
Q13- Do you eat dairy products (yogurt, milk, etc.) at breakfast?	0.234 (**0.023**)
Q14- Do you eat pastries or baked goods at breakfast?	0.218 (**0.035**)
Q15- Do you eat 2 yogurts and/or 40g of cheese per day?	0.067 (0.524)
Q16- Do you eat sweets or treats several times a day?	0.381(**<0.001**)
**Determinants for fast-food consumption**	
Sensory motivations (taste, toy)	0.035 (0.755)
Convenience motivations (price, quick service, lack of alternatives)	-0.117 (0.292)
Health motivations (health concerns, nutritional value)	-0.160 (0.149)

Values are presented as Spearman correlation coefficients, with corresponding p-values in parentheses.
